# Perinatal Supplementation with Omega-3 Polyunsaturated Fatty Acids Improves Sevoflurane-Induced Neurodegeneration and Memory Impairment in Neonatal Rats

**DOI:** 10.1371/journal.pone.0070645

**Published:** 2013-08-13

**Authors:** Xi Lei, Wenting Zhang, Tengyuan Liu, Hongyan Xiao, Weimin Liang, Weiliang Xia, Jun Zhang

**Affiliations:** 1 Department of Anesthesiology, Huashan Hospital, Fudan University, Shanghai, P. R. China; 2 National Key Laboratory of Medical neurobiology, Fudan University, Shanghai, P. R. China; 3 School of Biomedical Engineering and Med-X Research Institute, Shanghai Jiaotong University, Shanghai, P. R. China; Université Pierre et Marie Curie, France

## Abstract

**Objectives:**

To investigate if perinatal Omega-3 polyunsaturated fatty acids (n-3 PUFAs) supplementation can improve sevoflurane-induced neurotoxicity and cognitive impairment in neonatal rats.

**Methods:**

Female Sprague-Dawley rats (n = 3 each group) were treated with or without an n-3 PUFAs (fish oil) enriched diet from the second day of pregnancy to 14 days after parturition. The offspring rats (P7) were treated with six hours sevoflurane administration (one group without sevoflurane/prenatal n-3 PUFAs supplement as control). The 5-bromodeoxyuridine (Brdu) was injected intraperitoneally during and after sevoflurane anesthesia to assess dentate gyrus (DG) progenitor proliferation. Brain tissues were harvested and subjected to Western blot and immunohistochemistry respectively. Morris water maze spatial reference memory, fear conditioning, and Morris water maze memory consolidation were tested at P35, P63 and P70 (n = 9), respectively.

**Results:**

Six hours 3% sevoflurane administration increased the cleaved caspase-3 in the thalamus, parietal cortex but not hippocampus of neonatal rat brain. Sevoflurane anesthesia also decreased the neuronal precursor proliferation of DG in rat hippocampus. However, perinatal n-3 PUFAs supplement could decrease the cleaved caspase-3 in the cerebral cortex of neonatal rats, and mitigate the decrease in neuronal proliferation in their hippocampus. In neurobehavioral studies, compared with control and n-3 PUFAs supplement groups, we did not find significant spatial cognitive deficit and early long-term memory impairment in sevoflurane anesthetized neonatal rats at their adulthood. However, sevoflurane could impair the immediate fear response and working memory and short-term memory. And n-3 PUFAs could improve neurocognitive function in later life after neonatal sevoflurane exposure.

**Conclusion:**

Our study demonstrated that neonatal exposure to prolonged sevoflurane could impair the immediate fear response, working memory and short-term memory of rats at their adulthood, which may through inducing neuronal apoptosis and decreasing neurogenesis. However, these sevoflurane-induced unfavorable neuronal effects can be mitigated by perinatal n-3 PUFAs supplementation.

## Introduction

Sevoflurane is one of the most frequently used volatile general anesthetic agents used during surgical procedures. It is especially useful for pediatric anesthesia because sevoflurane allows rapid induction and recovery and is less irritating to the airway than other inhaled anesthetics [1]. Recent evidence demonstrates that volatile anesthetics can induce neuronal apoptosis [2], [3], affect neurogenesis *in vitro* and *in vivo* [4], and disturb long-term neurocognitive function in 7-day-old rats [5]. Although several studies report sevoflurane is less cytotoxic than isoflurane and desflurane, sevoflurane exposure in neonates reportedly increases risk for neurodevelopmental impairments in animal models [6]–[9]. Given its clinical relevance and potential for unfavorable outcomes in pediatric anesthesia, we sought to substantiate sevoflurane's putative neurotoxic effects, and develop a strategy to prevent sevoflurane-induced neurodevelopmental impairment in neonatal rats.

Omega-3 polyunsaturated fatty acids (n-3 PUFAs) are essential dietary nutrients that play critical roles in brain development and function. Their contributions to learning and memory are well documented with maternal n-3 PUFA supplementation during gestation [10]. Prenatal n-3 PUFAs supplementation confers long-term neuroprotection against neonatal hypoxia-ischemic injury via anti-inflammatory actions [11], and attenuates hyperoxia-induced neuronal apoptosis in the developing brain [12]. Conversely, n-3 PUFAs deficiency altered neurogenesis in embryonic [13] and adult [14] rat brains. They also may exert effects in human neurodegenerative conditions. In a randomized double-blind trial, n-3 PUFAs administration demonstrated positive effects in a small group of Alzheimer's patients [15]. The effect of n-3 PUFAs on postnatal anesthetic-induced neurotoxicity in the developing brain, however, has never been studied. We hypothesized that n-3 PUFAs supplementation during pregnancy and lactation could protect against neurotoxicity in neonatal rats exposed to sevoflurane anesthesia.

## Materials and Methods

### Animal/Anesthesia treatment

The rats used in the present study were obtained from the Animal Care Center of Fudan University. The study protocol was reviewed and approved by the Institutional Animal Care and Use Committee, Fudan University. One-day pregnant female Sprague Dawley rats (weight 220–250 g) were randomly assigned to one of the three groups: control, sevoflurane, or sevoflurane with n-3 PUFAs (n = 3 per group). Fish oil, the main source of n-3 PUFAs (Eicosapentaenoic acid (EPA), docosahexaenoic acid (DHA)), was extracted from the capsule (1000 mg/capsule that containing 180 mg EPA and 120 mg DHA, Puritan's Pride, Bohemia, NY, USA) and added to food. Pregnant dams in the sevoflurane and control groups were fed a regular laboratory rodent diet with a low n-3 PUFAs concentration (0.5% of total fatty acid), whereas the sevoflurane with n-3 PUFAs group were fed the same diet, but supplemented with n-3 PUFAs (15 mg fish oil/g regular diet) from day 2 of pregnancy to 14 days after parturition. Dams were given free access to food and water, and dams for all groups were kept under identical housing conditions with a 12-h light cycle. On postnatal day 7 (P7), the rat pups in the sevoflurane and sevoflurane with n-3 PUFAs groups received sevoflurane anesthesia.

**Figure 1:**
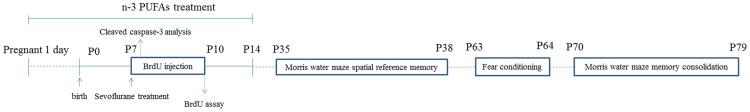
Schematic timeline of the experimental procedure. Omega-3 polyunsaturated fatty acids (n-3 PUFAs) supplementation began in dams from pregnancy day 2 to 14 days after parturition or until the day when the brains of their offspring were harvested. The neonatal rats were exposed to 3% sevoflurane (Sevo) for 6 h at their seventh day (P7). The behavioral tests including Morris water maze spatial reference memory, fear conditioning, and Morris water maze memory consolidation on P35–38, P63–64, and P70–79, respectively. P = postnatal day.

P7 rats were placed in a sealed box ventilated with 3% sevoflurane in 60% oxygen and treated for 6 h. The temperature in the sealed box was maintained at 33–35°C. The total survival percentage of P7 rats after 6-h anesthesia was 88.4%; the likely cause of death was respiration depression. After anesthesia, the pups were returned to the dams. Control rat pups were placed in the same box without sevoflurane exposure and under identical experimental conditions. The flow chart for the experimental protocol is summarized in Figure 1.

### Blood gas analysis

Twelve naïve P7 rats that that did not participate in other experiments were used to assess the effect of sevoflurane on blood gases. Blood was percutaneously aspirated from the left cardiac ventricle after 0, 2, 4, and 6 h of anesthesia (n = 3 per time point). From these samples, we measured partial pressures of carbon dioxide and oxygen, pH, and blood lactate and glucose levels with a Radiometer ABL 800 blood gas analyzer (Radiometer, Copenhagen, Denmark).

### BrdU injection

To determine whether sevoflurane affects progenitor cell proliferation in the S-phase of the cell cycle, bromodeoxyuridine ((+)-5′-bromo-2′-deoxyuridine [BrdU]; 97%; Sigma-Aldrich, St. Louis, MO, USA) in 0.9% sterile saline solution was injected intraperitoneally using the procedure described by Wojtowicz [16]. The first dose (150 mg/kg) was administered immediately before sevoflurane treatment, and the three subsequent injections (50 mg/kg BrdU) were given at 24-h intervals following sevoflurane anesthesia.

### Tissue preparation and immunohistochemistry

Animals were deeply anesthetized with chloral hydrate and then transcardially perfused with 0.9% saline followed by 4% paraformaldehyde in 0.1 M phosphate buffered saline (PBS), pH 7.4. The brains were removed, postfixed overnight in 4% paraformaldehyde/PBS, and placed in 30% sucrose until they sank in the solution. Coronal sections (30 µm) were cut on a microtome (Leica CM1900 UV, Wetzlar, Germany) and every sixth section was stored in 30% sucrose containing 30% ethylene glycol to stain BrdU or cleaved caspase-3.

For immunocytochemical detection of BrdU-labeled nuclei, DNA was denatured to expose the antigen for incubation with 2 N hydrochloric acid for 30 min at 37°C, followed by neutralization with two 10-min incubation periods in 0.5 M boric acid (pH 8.5) at room temperature (RT). Sections were subjected to three 10-min washes in PBS with 0.3% Triton-X with 10 min between each wash. Nonspecific epitopes were blocked with 1% serum for 30 min at RT, and were incubated overnight at 4°C with either BrdU (1∶100; BD Pharmingen, Franklin Lakes, NJ, USA) or cleaved caspase-3 (1∶1,000; Cell Signaling, Danvers, MA, USA) antibody in PBS and 1% serum. On day 2, the sections were incubated with the appropriate secondary fluorescent antibodies (Alexa Fluor 488, 1∶200; Invitrogen, Carlsbad, CA, USA) for 2 h at RT, followed by three 5-min washes in PBS. Nuclear counterstaining was performed with 4′,6-diamidino-2-phenylindole (1∶500; Beyotime Institute of Biotechnology, Haimen, China), which was followed by mounting and coverslipping with an aqueous mounting medium. Images were acquired with a microscope (Leica DM2500). BrdU- or cleaved caspase-3-positive cells were counted in a blinded manner at ×20 magnification [17]. Questionable structures were excluded from the count if their identification remained uncertain under ×40 magnification.

### Western blot analysis

The cerebral cortex, thalamus, and hippocampus were harvested 18 h after sevoflurane treatment. The brain tissues were homogenized in RIPA buffer (Millipore, Temecula, CA, USA) containing complete protease inhibitor cocktail and 2 mM phenylmethylsulfonyl fluoride. The lysates were collected and centrifuged at 12,000 rpm for 30 min at 4°C. After the protein samples were quantified using a BCA Protein Assay Kit (Pierce Biotechnology, Rockford, IL, USA), 60 µg of each sample was electrophoresed through a 14% sodium dodecyl sulfate-polyacrylamide gel and wet electrotransferred to 0.45-µm nitrocellulose membranes (Millipore). The blots were incubated overnight at 4°C with a polyclonal anti-cleaved caspase-3 antibody, and then incubated with a rabbit anti-mouse polyclonal horseradish peroxidase-conjugated secondary antibody (1∶5,000; Epitomics, Hangzhou, Zhejiang Province, China) at RT for 1 h. Protein signals were detected using an enhanced chemiluminescence detection system (Pierce Biotechnology). A β-actin antibody (1∶1,000; Santa Cruz Biotechnology, Santa Cruz, CA, USA) was used to normalize sample loading and transfer. Band intensities were densitometrically quantified using Gel-Pro Analyzer (Media Cybernetics, Bethesda, MD, USA).

### Neurobehavioral tests

**Figure 2:**
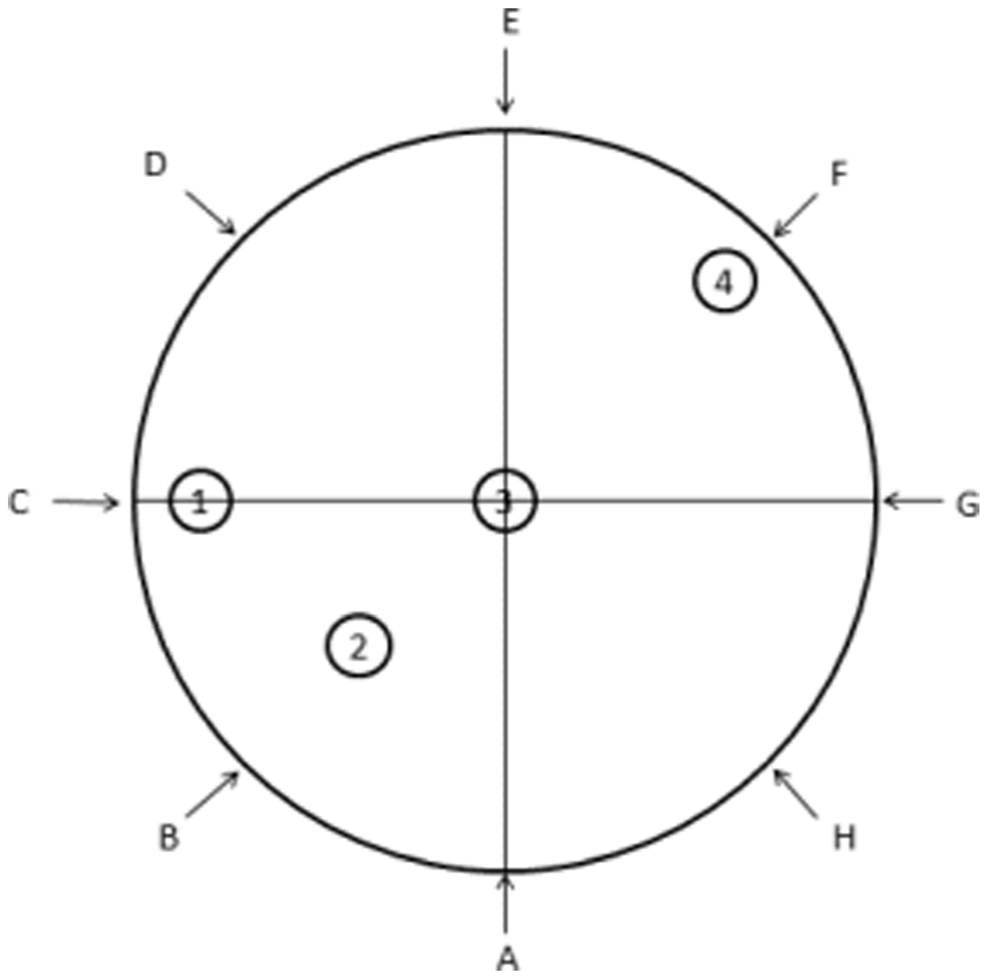
Morris water maze setup. Numbers: platform location; Letters: drop location. In the spatial reference memory task, the platform location is in the middle of one of four virtual quadrants (2). In the probe training session, the rat are released from four pseudorandomly assigned points (D,E,H and G) which provide two short and two medium swims to the platform location per session. In the probe test session, the drop location (F) is at the opposite of original platform. In the memory consolidation task, the platform location are quarter-way between the center of the maze and the wall of the tank on the border of two quadrants (1) or within a quadrant (4), or in the center of the maze (3) or in the middle of one of four virtual quadrants (2). The drop location was pseudorandomly varied to incorporate one short, one medium, and one long swim to platform.

We used only male offspring (n = 9 per group) in the neurobehavioral tests to exclude estrogen influences on neurocognitive evaluations. The water maze setup in spatial reference memory task and memory consolidation task was shown in Figure 2.

#### Morris water maze spatial reference memory

*Probe training:* Rats trained for 4 consecutive days (postnatal days 35–38, P35–38) in the Morris water maze following treatment with a vehicle or 3% sevoflurane for 6 h. A platform (10.3-cm diameter) was submerged in a circular pool (180-cm diameter, 50-cm depth) filled with warm (23–25°C) opaque water. Rats performed two training sessions each day. In each session, rats performed four trials in which they were released from one of four pseudorandomly assigned release points while facing the tank wall. This provided two short and two medium swims per session. Animals were allowed 60 s to locate the hidden platform, and if they failed to find the hidden platform in the allotted time, the investigator guided the animal to the platform. In either case, the rats were removed from the platform after 15 s. Training sessions were conducted until the rats could locate the hidden platform in less than 15 s in at least five sessions (average time per session). All trials were videotaped, and rat swim paths were recorded with ANY-maze video tracking system (Stoelting Co., Wood Dale, IL, USA), which allowed us to measure the time taken (latency) to find the platform(s), as well as other behavioral information obtained during the spatial reference memory test. The animals were dried and placed beneath a heating lamp after completing each test.

*Probe test*: A probe trial was performed with the platform removed from the tank to assess memory retention for the hidden platform location. Probe trials were administered 1 day after the last training session (P38). During the 60-s probe trial, we determined the number of entries into the platform quadrant zone, the swimming speed (cm/s), the total distance (cm), and the time spent in the target quadrant relative *versus* the other quadrants.

#### Fear conditioning test

Rats underwent fear conditioning tests on postnatal days 63–64 (P63–64). Every time four rats randomly chosen from three groups were trained in each session. Rats were placed in plastic chambers with a grid floor constructed from 19 stainless steel bars (4-mm diameter, spaced every 16 mm). The floors were connected to a shock delivery system (Coulbourn, Whitehall, PA, USA), and electrical shocks were delivered through the stainless steel bars. The chamber was illuminated with overhead fluorescent bulbs, and a ventilation fan provided background noise (65 db). The training context was considered the appearance, odor, and texture of the environment (chamber and room) in which the rats were trained. After a 3-min baseline exploratory period, rats were presented with three auditory tones (2,000 Hz, 90 db) that were followed 1 min later by an electric shock (1 mA, 2 s). We quantified the rats' fear response with freezing, which is an innate defensive fear response in rodents and a reliable measure of learned fear. Freezing was defined as the lack of movement, except for respiration. We examined rats in the fear condition test the day after they first received the electrical shock to determine whether they showed fear to the training context or the auditory tone. For the context test, rats were placed in the chamber where they were trained on the previous day. The rats remained in the chamber for 8 min, without an auditory tone or shock. For the tone test, rats were transported in groups to a context chamber with black boards covering the walls. Rats were allowed a 3-min exploratory period before three 30-s tones were played (2,000 Hz, 90 db, separated by 60 s). Rats were removed from the chamber 30 s after the tone presentation. The order of the context and tone tests was counterbalanced so that half of each treatment group first was tested for context and then for tone, whereas the other half of the treatment group was tested in the reverse order. FreezeView software (Coulbourn) was used to score each animal's freezing behavior separately for the training period and the context and tone tests, which were expressed as a percentage.

#### Morris water maze memory consolidation

*Working memory (WM)*: On postnatal day 70 (P70), the testing room was rearranged by repositioning the water tank and adding new spatial cues. The platform was submerged 1.5 cm below the water surface in one of four designated platform positions. From P70 onward, one session was conducted per day. Each session began with a 60-s free swim (performance not scored) in which rats explored the maze, and was followed by a 1-min rest interval and three subsequent scored trials. Rats that found the platform during the free swim were allowed to rest on the platform for 15 s. Rats that failed to find the platform during the free swim were guided to the platform and remained there for 15 s. After the free swim, three trials were administered in which the rat was released from one of six pseudorandomly chosen locations that faced the tank wall. The platform location was identical for all animals in a session, but the drop location was pseudorandomly varied to incorporate one short, one medium, and one long swim. Training sessions were administered until the session average for finding the hidden platform was less than 15 s. The latency for reaching the platform was recorded by the ANY-maze video tracking system.

*Short-term memory (STM) and early long-term memory (ELTM):* When the WM latencies of rats in task were plateaued on postnatal day 77 (P77), we increased the delay between the free swim and the subsequent trials. The delay was extended from 1 min on P77 to 1 h on postnatal day 78 (P78) to test STM, and then to 4 h on P79 to test ELTM. Performances on the last trial after the free swim on P77 (1-min delay), P78 (1-h delay), and P79 (4-h delay) were used as measures of WM, STM, and ELTM, respectively.

### Statistical methods

All data are presented as mean ± standard deviation. We performed two-tailed *t* tests (assuming equal variances) to determine differences in cleaved caspase-3 immunohistochemistry and blood gas parameters between the control and sevoflurane groups. We used a one-way analysis of variance followed by Newman-Keuls post hoc tests to determine differences among groups for interactions between n-3 PUFAs or sevoflurane and cleaved caspase-3 activation, BrdU quantification, or neurobehavioral tests. For all tests, *p*<0.05 was considered statistically significant.

## Results

### Blood gas analysis

**Figure 3:**
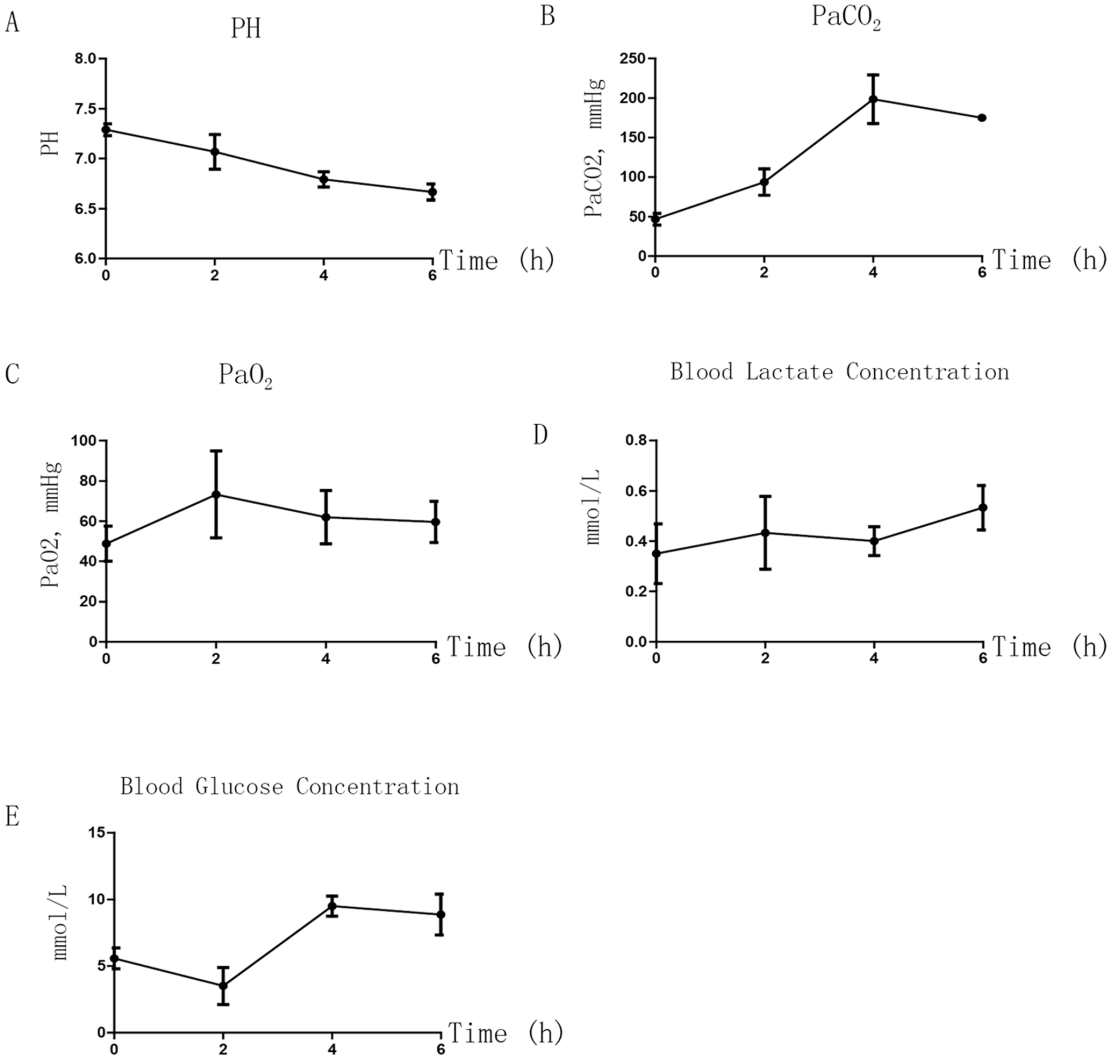
Arterial blood gas and biochemical analysis. A: pH; B: PaCO_2_; C: PaO_2_; D: Blood lactate concentration; E: Blood glucose concentration. FiO_2_: fraction of inspired oxygen; PaCO_2_: partial pressure of carbon dioxide; PaO_2_: partial pressure of oxygen. Sevo treatment could decrease pH (A) due to hypercarbia (B) and increase the blood glucose concentration; n = 3 at each time.

We assessed blood gas and biochemical changes in P7 rats treated with continuous 3% sevoflurane in 60% oxygen for 0, 2, 4, or 6 h. The partial pressures of carbon dioxide and oxygen, pH, and blood glucose and lactate levels at each time point are shown in Figure 3. We observed that prolonged sevoflurane anesthesia caused hypercarbia, but not hypoxemia, which could be due to respiration depression.

### Neuronal apoptosis

**Figure 4:**
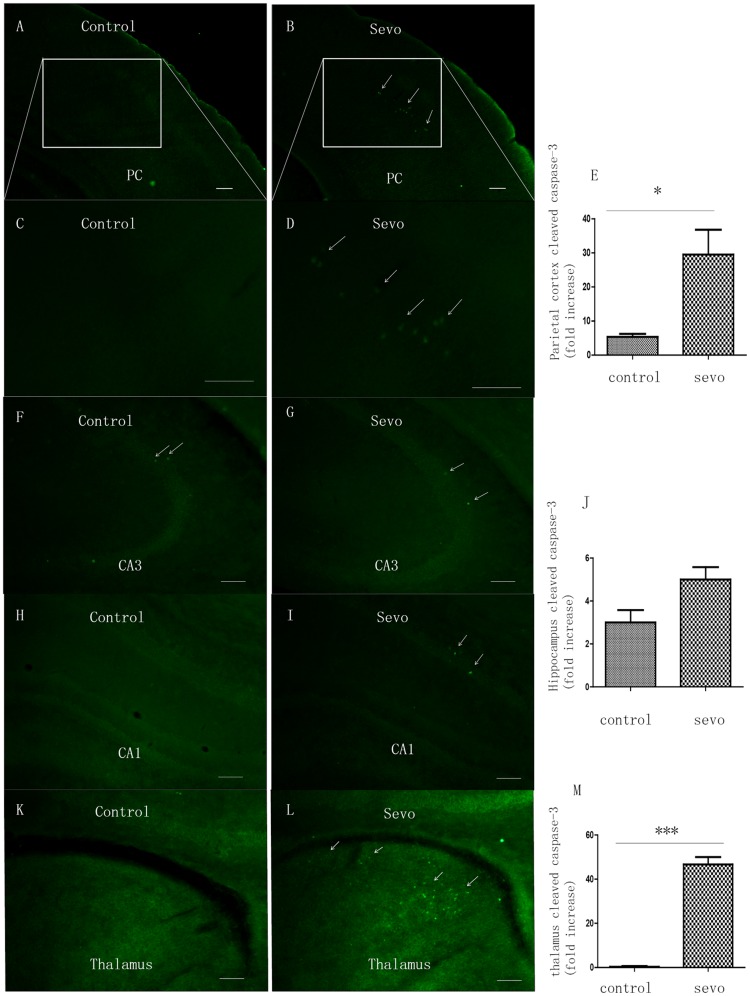
The effects of neonatal sevoflurane exposure on caspase-3 expression. Immunofluorescence revealed the effects of the 6-h 3% sevoflurane exposure on cleaved caspase-3 expression in neonatal rat brains at P7 (n = 3 in each group). The photomicrographs (5×) of cleaved caspase-3 in the parietal cortex in control group (A) and in the Sevo group (B); C: The photomicrographs (5× with 20× inset) of cleaved caspase-3 in the parietal cortex in control group (C) and in the Sevo group (D); Quantification of cleaved caspase-3 in parietal cortex (control vs. Sevo, *p* = 0.0389) (E); The photomicrographs (10×) of cleaved caspase-3 in the CA1 (F), CA3 (H) of controls and CA1 (G), CA3 (I) in Sevo group; Quantification of cleaved caspase-3 in hippocampus region (control vs. Sevo, NS) (J); Photomicrographs (10×) of cleaved caspase-3 in the thalamus in control group (K) and in Sevo group (L); Quantification of cleaved caspase-3 in the thalamus region (control vs. Sevo, *p* = 0.0002) (M).

Caspase-3 is a ubiquitously distributed caspase, and its activation strongly suggests cellular apoptosis [18]. Eighteen hours after sevoflurane anesthesia induction, neonatal rats showed greater amounts of cleaved caspase-3 immunoreactivity in the parietal cortex (Figure 4A–E) and thalamus (Figure 4K–M) compared to controls. Our results indicate that sevoflurane dramatically increased the incidence of apoptosis in thalamic neurons (median 46-fold increase compared with control). Although sevoflurane treatment slightly increased apoptosis in cornu ammonis (CA) 1 and 3 hippocampal regions (Figure 4F–J), these changes were not significant.

### n-3 PUFAs attenuate sevoflurane-induced neuronal apoptosis

**Figure 5:**
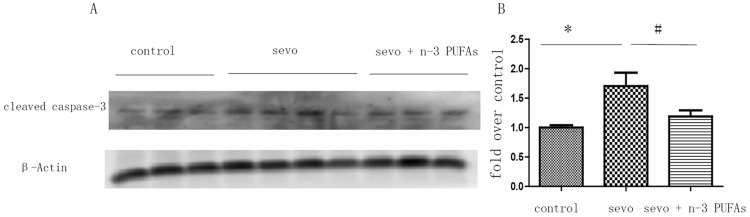
Perinatal n-3 PUFAs supplementation attenuates 6-h 3% sevoflurane-induced neuronal apoptosis. Cortical cleaved caspase-3 expression in neonatal brain was examined with Western blot (A); Quantification of cleaved caspase-3 (one-way ANOVA, Newman-Keul post hoc test, F = 6.286, *p* = 0.0274), *p<0.05 control (n = 3) vs. Sevo (n = 3); #*p*<0.05 Sevo vs. Sevo+n-3 PUFAs (n = 4) (B).

We examined cortical extracts with western blots to verify activated caspase-3 immunofluorescence represented apoptosis, and to quantify the apoptotic response. Cleaved caspase-3 immunoblotting confirmed that 3% sevoflurane treatment for 6 h increased caspase-3 activity in the parietal cortex of neonatal rat pups. Perinatal n-3 PUFAs supplementation significantly ameliorated cleaved caspase-3 expression in the parietal cortex (Figure 5) of offspring rats that underwent sevoflurane anesthesia.

### n-3 PUFAs reverse sevoflurane-induced inhibition of hippocampal neuronal proliferation

**Figure 6:**
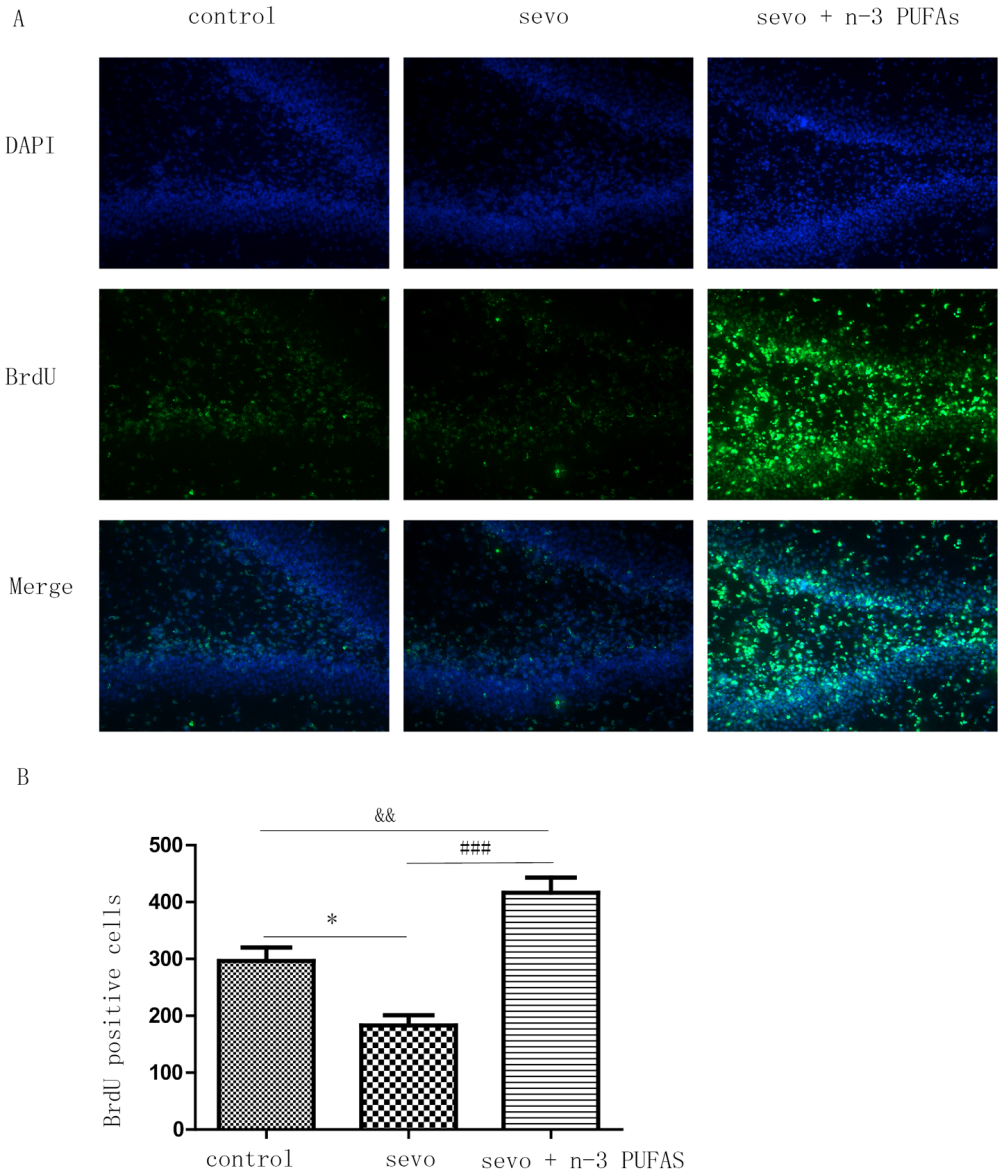
Perinatal n-3 PUFAs supplementation increases the attenuation of neuronal cells proliferation caused by neonatal exposure to 3% sevoflurane for 6-h in dentate gyrus (DG) region of neonatal hippocampus. BrdU was examined at the DG region by immunofluorescence (A); Quantification of BrdU at the DG region of neonatal hippocampus (one-way ANOVA, Newman-Keul post hoc test, F = 26.07, *p* = 0.0011), *p<0.05 control vs. Sevo; ###*p*<0.05 Sevo vs. Sevo+n-3 PUFAs; &&*p*<0.01 control vs. Sevo+n-3 PUFAs (B). n = 3 in each group.

Newly generated BrdU-labeled cells were observed in the dentate gyrus (DG) subgranular zone following immunofluorescence labeling. Sevoflurane decreased the number and fluorescence intensity of BrdU-labeled cells in the DG compared with controls, but these changes were attenuated by perinatal n-3 PUFAs supplementation (Figure 6). These findings suggest that perinatal n-3 PUFAs supplementation can reverse sevoflurane-induced neuronal proliferation inhibition in neonatal rat hippocampus.

### n-3 PUFAs improve neonatal sevoflurane-induced neurobehavioral deficits in adulthood

**Figure 7:**
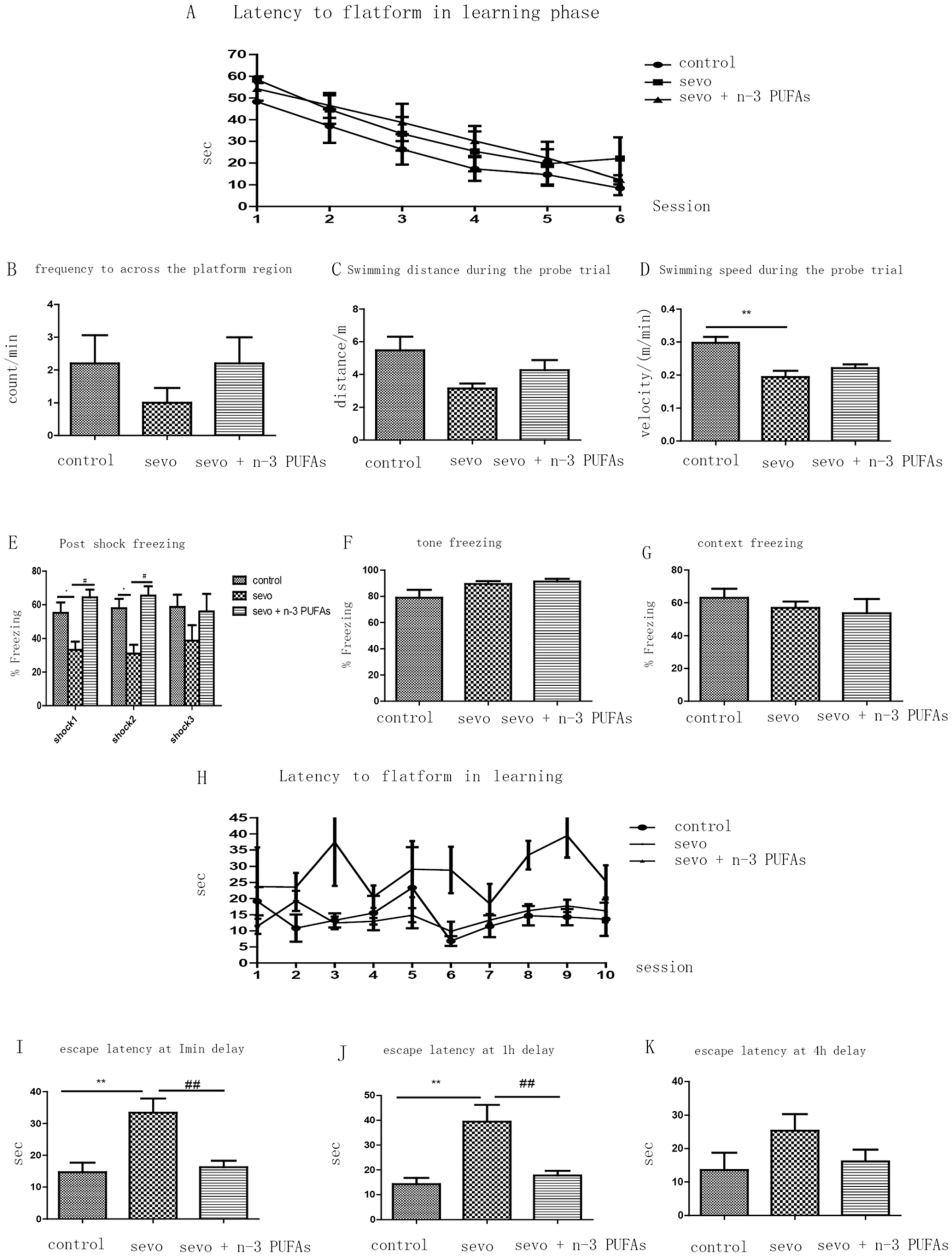
Perinatal n-3 PUFAs supplementation improves neonatal sevoflurane exposure induced neurobehavioral impairment at adulthood (n = 9 each group). A–D Morris water maze spatial reference memory. Latency to platform in learning phase (A); Frequency to across the platform region (B); Swimming distance during the probe trial (C); Swimming speed during the probe trial (D); **p = 0.0019 control vs. Sevo. Fear conditioning (E–G). Post shock freezing (E): post shock 1 (F = 29.437, *p* = 0.0041, one-way ANOVA, Newman-Keul post hoc test, *p<0.05 control vs. Sevo; #*p*<0.05 Sevo vs. Sevo+n-3 PUFAs); post shock 2 (F = 10.3, *p* = 0.0033, one-way ANOVA, Newman-Keul post hoc test, *p<0.05 control vs. Sevo group; #*p*<0.05 Sevo vs. Sevo+n-3 PUFAs). Tone freezing (F); Context freezing (G); Morris water maze memory consolidation (H–K): Latency to platform in learning phase (H); Escape latency at 1-min delay (I) F = 10.25, *p* = 0.0031, one-way ANOVA, Newman-Keul post hoc test,**p<0.01 control vs. Sevo, ##*p*<0.01 Sevo vs. Sevo+n-3 PUFAs; Escape latency at 1-h delay (J); F = 13.70, *p* = 0.0014, one-way ANOVA, Newman-Keul post hoc test, **p<0.01 control vs. Sevo ##*p*<0.01 Sevo vs. Sevo+n-3 PUFAs; Escape latency at 4-h delay (K).

Sevoflurane anesthesia and n-3 PUFAs supplementation did not affect escape latencies during the six probe training sessions in the Morris water maze (Figure 7A). Similarly, sevoflurane anesthesia did not affect the frequency required to cross the platform region (Figure 7B) or the swimming distance (Figure 7C) during the probe trial. Finally, n-3 PUFAs supplementation did not affect the sevoflurane-induced reduction in swimming speed (Figure 7D).

In the fear conditioning training session, the postshock freezing response in rats with neonatal exposure to sevoflurane was significantly decreased compared with controls for shocks 1 and 2. Maternal n-3 PUFAs supplementation, however, significantly increased postshock freezing in the offspring. Postshock freezing was similar across all groups for the third tone/shock pairing (Figure 7E). To assess the influence of neonatal exposure to sevoflurane on ELTM, rats underwent contextual/cued fear conditioning tests. In this paradigm, we failed to find any differences after one training day (Figure 7F–G). These results indicate that sevoflurane has no effect, and n-3 PUFAs did not further improve ELTM in the fear conditioning test.

Memory consolidation describes the transition from unstable memories to stable memories. At least four different stages of memory consolidation are distinguished, three of which are assessed here: WM (minutes), STM (minutes to hours), and ELTM (greater than 3 h). We did not assess remote long-term memory because this process requires delays between memory formation and recall that span weeks to months. In the 1-min delay training session, sevoflurane increased escape latencies in the sixth and eighth sessions, whereas fish oil supplementation decreased the escape latencies in sevoflurane-treated animals in the fourth, sixth, and eighth sessions (Figure 7H–I). In the 1-h delay session (session 9), sevoflurane-treated rats had significantly longer escape latencies than controls, and sevoflurane-treated rats pre-treated with fish oil had significantly lower escape latencies than sevoflurane-treated rats (Figure 7J). Sevoflurane anesthesia impaired performance in a spatial recognition memory task when 1-min and 1-h delays were introduced between memory encoding and memory retrieval, and perinatal n-3 PUFAs supplementation alleviated this deficit. These results indicate that sevoflurane can impair WM and STM, but n-3 PUFAs can alleviate these impairments. Interestingly, there was no significant difference among groups in escape latency at the 4-h delay session (session 10, Figure 7K), indicating that sevoflurane may not affect ELTM.

## Discussion

### Main findings

The major findings in this study were as follows: (1) sevoflurane exposure induced neuronal apoptosis in rat pups, (2) sevoflurane exposure decreased hippocampal neuron proliferation in neonates, (3) sevoflurane exposure in neonates impaired STM two months later, and (4) perinatal n-3 PUFA supplementation protects neurons against all three of these sevoflurane-induced changes.

#### Effects of sevoflurane anesthesia on neuronal apoptosis

Compared with littermate controls, rat pups exposed to 3% sevoflurane for 6 h on P7 (third trimester-equivalent in humans) showed increased apoptotic neurodegeneration in major brain regions that are important for learning and memory. Neuronal apoptosis was partly attributed to hypercarbia caused by sevoflurane-induced respiratory depression, although a previous report indicated that hypercarbia does not cause significant neurocognitive impairment [19]. Some studies indicate sevoflurane can induce neuronal apoptosis in neonatal rodents, but most of these studies examined apoptosis only in neocortical [20], [21] or hippocampal [22] tissue. We found that neonatal sevoflurane exposure induced caspase-3 activation in the cortex and thalamus, but not the hippocampus. Similarly, Zhu et al. [4] reported that isoflurane had no obvious effect on hippocampal cell death in P14 rats. Anesthetic treatment therefore may have differential effects on neurons at various developmental stages.

#### Effects of sevoflurane anesthesia on neurogenesis

Neonatal neurogenesis begins when cells proliferate and ends when cells migrate and integrate into a neuronal circuit as a functional neuron. It is widely believed that neurogenesis enables hippocampal plasticity and new memories [23]. In addition to neuronal apoptosis, several recent studies correlate alterations in neurogenesis with cognitive performance [24], [25]. Hippocampal neurogenesis is initiated after volatile anesthesia in P7 rats [26]–[28], and the mechanisms underlying this phenomenon have been reviewed [29]. The effect of general anesthesia on neurogenesis, however, is controversial. Whether anesthesia stimulates or depresses neurogenesis appears to depend on the duration, concentration [30], and type of anesthetic administrated [31], [32], as well as the animal model used [27] and the experimental conditions [33]. We found that 3% sevoflurane treatment for 6 h significantly decreased the number of BrdU^+^ cells in the hippocampus DG in P7 rats. Because this inhibition of proliferation can persist for more than 4 weeks, the neural progenitor pool in DG can be greatly reduced and affect subsequent neurogenesis [4]. Why sevoflurane anesthesia influences proliferation without causing neuronal apoptosis in the developing hippocampus is unknown. Nevertheless, the sensitivity of the neonatal central nervous system to sevoflurane may affect brain areas differently, depending on the survival, proliferation, differentiation, and migration patterns of neurons for each region.

### Effects of sevoflurane anesthesia on learning and memory

Both juvenile and adult rodents exposed to volatile anesthetics during gestational or neonatal development can show learning and memory impairments [6], [34]. We employed a series of neurobehavioral tests and also found that sevoflurane exposure in neonates resulted in long-term neurocognitive sequelae. Interestingly, we did not observe significant effects on traditional “hippocampus-dependent” ELTM 8 weeks after sevoflurane anesthesia, but sevoflurane did significantly reduce the immediate fear response to the tone/shock pairings in the fear conditioning test. In addition, sevoflurane impaired spatial memory in the Morris water maze memory consolidation test when the delay between memory acquisition and retrieval was extended from 1 min to 1 h, but not to 4 h. This suggests that sevoflurane impaired WM and STM, rather than ELTM. Collectively, these neurobehavioral tests suggest that sevoflurane has negative effects on WM and STM, but not ELTM. Conversely, Kodama et al. [8] demonstrated that sevoflurane treatment did not impair WM in neonates. The brain regions that were most affected by neuronal apoptosis, changes in memory-related signaling [35], and protein production [36] with sevoflurane exposure are important for learning and memory, which may explain this discrepancy between the results from Kodama et al and ours [37]. Although we examined three brain regions that play major roles in the early stages of learning [38] and the three types of memory [39], other regions also participate in these cognitive functions. Shih et al [7] attributed neurocognitive dysfunction to acute sevoflurane-induced neuron death, especially in the thalamus, which was the most severely affected region. In that study, however, the authors did not examine the effect of anesthesia on neurogenesis in the neonatal brain. Consistent with the findings in previous studies, we found that sevoflurane decreased neuronal proliferation in the developing hippocampus. Similarly, a previous study reported that inhibition of neurogenesis interfered with memory function [40]. We speculate that sevoflurane-induced neuron death and failed proliferation in the developing central nervous system reduce brain volume and the number of synaptic connections, and disrupts brain plasticity, which is similar to the effects of alcohol on brain [41]. Neurons lost to apoptosis can be compensated via neurogenesis, as has been reported in stroke cases [42]. However, it is more essential that these newly generated neurons can integrate into an existing brain network and function in learning and memory.

#### n-3 PUFAs protect against sevoflurane-induced neurotoxicity and behavioral deficits

Considering volatile anesthetics can impair brain function in rodents, finding interventions that improve or prevent sevoflurane-induced memory deficits in the developing brain might provide insight into the neurotoxic mechanisms of volatile anesthetics. Although some drugs can protect against anesthesia-induced neurotoxicity [43], n-3 PUFAs commonly have been used as a daily supplement for pregnant and lactating women as it benefits neurodevelopmental outcomes [44]. Therefore, we decided to test whether n-3 PUFAs could improve sevoflurane-induced brain dysfunction. Our data revealed that n-3 PUFA dietary supplementation to dams led to significant and prolonged neuroprotection in offspring rats that received neonatal sevoflurane exposure. This is the first report of n-3 PUFAs exerting protective effects against sevoflurane-induced cognitive impairment in rats, specifically by attenuating apoptosis and improving neuronal proliferation.

Furthermore, there were significantly more BrdU^+^ immunoreactive cells in the hippocampus DG when n-3 PUFA supplementation was combined with sevoflurane anesthesia, than with sevoflurane anesthesia alone. Compared with the control group, the robust effect of n-3 PUFAs on neurogenesis does not appear to promote performance improvements in the neurobehavioral tests. One possible explanation for this observation is that the proliferative cells do not survive [45], differentiate into mature neurons, or effectively integrate into circuits for learning and memory. A longer observational period is needed to assess these possibilities.

Although we show that n-3 PUFAs have anti-apoptotic effects and neurogenesis-promoting properties, the molecular signaling involved in these mechanisms are unclear. Zhang and coworkers showed that n-3 PUFAs could confer long-term neuroprotection against hypoxic-ischemic brain injury by suppressing the inflammatory response [11], and proinflammatory factors are believed to be one of the pro-apoptosis factors that contributes to volatile anesthetic-induced neurotoxicity [46]. In addition, maternal feeding of DHA significantly prevented stress-induced oxidative damage, apoptosis, and mitochondrial metabolism dysfunction in the hippocampus of offspring [47]. Wu et al [48] found that dietary n-3 PUFAs could normalize brain-derived neurotrophic factor (BDNF) levels in a rat model of traumatic brain injury, which is important for neuronal survival, differentiation, and function. It is possible that maternal n-3 PUFA supplementation during pregnancy could protect against postnatal reduction of brain neurotrophins in offspring [49]. Thus, n-3 PUFAs could alleviate neuronal apoptosis via regulating the inflammatory response, restoring BDNF imbalance [50], and/or decreasing reactive oxygen species levels [51] induced by sevoflurane in developing neurons. On the other hand, neurogenesis impairment might be caused by an anesthetic-induced decrease in trophic support (e.g., reduced BDNF levels), and maternal n-3 PUFA supplementation during pregnancy can protect against postnatal reduction of brain neurotrophins (BDNF and nerve growth factor) in offspring [52], which may promote neurogenesis in the developing brain. Collectively, these data support the hypothesis that n-3 PUFAs make several important contributions to the developing nervous system that help to prevent learning and memory impairments [11], [48], [53].

### Limitations

There were some limitations to the present study. Firstly, we did not include an experimental group that received n-3 PUFAs during the perinatal period, without sevoflurane exposure. Nevertheless, our primary aim was to determine whether n-3 PUFAs mitigated sevoflurane-induced neurotoxicity and subsequent cognitive impairment, rather than determine the effects of n-3 PUFAs themselves. Secondly, we did not measure the fatty acid contents in brain and plasma of the rats at several stages (e.g., P0, P14, P38, P64, P79). In a previous study, we have found perinatal n-3 PUFAs supplementation increased functional polyunsaturated fatty acid composition in brain cortical tissue in neonatal rats at postnatal 14 day with the same dietary feeding protocol used in this study [11]. The results from that study suggest increased n-3PUFAs levels in the brain at P14 are important for neuroprotection in the perinatal period, and later in life. Finally, we did not investigate whether the number of immature neurons was affected by postnatal sevoflurane exposure in P7–10 male rats. Even so, 80–90% of proliferating cells in the hippocampus DG differentiated into mature neurons; therefore, a decrease in BrdU^+^ cell numbers suggest neonatal sevoflurane exposure influences hippocampal neurogenesis in postnatal rats at P7–10. A recent study showed that exposure to general anesthetics during development appears to influence the percentage of neurons in a new cell population [54]. Therefore, future work should examine which cell types are affected by sevoflurane anesthesia by immunolabeling with specific neuronal cell markers.

### Conclusion

Sevoflurane exposure in neonates results in neuronal apoptosis and impaired proliferation, both of which can cause neurocognitive disabilities later in life. In addition, our results provide evidence that perinatal n-3 PUFA supplementation can improve neurocognitive deficits, possibly by reducing neuronal apoptosis and neurogenesis impairment in the developing brain. Nevertheless, it is critical to recognize that rodent brain development is fundamentally different from that of humans, and the present results might not be directly translated into clinical practice. Hence, further investigations are warranted to fully understand the effects of n-3 PUFAs on general anesthetic-induced neurotoxicity.
